# High-efficiency removal of methyl orange from wastewaters using polyimide/chitosan-MoS_2_-UiO-66 nanofiber adsorbents

**DOI:** 10.1038/s41598-025-32760-x

**Published:** 2025-12-17

**Authors:** Vahid Hemmati, Gholamreza Karimi, Dariush Mowla

**Affiliations:** 1 Chemical Engineering Department, School of Chemical and Petroleum Engineering, Shiraz University, Shiraz, 7134851154 Iran; 2 Environmental Research Center in Petroleum and Petrochemical Industries, School of Chemical and Petroleum Engineering, Shiraz University, Shiraz, 7134851154 Iran

**Keywords:** Methyl orange, Dye removal, Polyimide nanofibers, Chitosan, MoS_2_, UiO-66, Chemistry, Environmental sciences, Materials science, Nanoscience and technology

## Abstract

Conventional adsorbents such as chitosan and layered MoS_2_ show promising functional groups or surface activity but suffer from intrinsic drawbacks such as limited surface area, and nanosheet aggregation, which restrict their efficiency in rapid dye removal. To overcome these limitations, we designed polyimide/chitosan-MoS_2_-UiO-66 (PI-CS-MoS_2_-UiO-66) nanofibrous adsorbents that integrate structural stability, abundant functional groups, and high porosity within a single hybrid system. The nanofibers were prepared by electrospinning PI as a robust support, followed by dip-coating with CS-MoS_2_-UiO-66 and crosslinking. XRD, BET, FT-IR, TGA, and SEM confirmed the successful incorporation of the composite. Adsorption tests on methyl orange (MO) showed strong dependence on pH, dosage, initial concentration, and temperature. The maximum adsorption capacity reached 195.61 $\:\frac{\mathrm{m}\mathrm{g}}{\mathrm{g}}$ with excellent correlation to the Langmuir isotherm (R^2^ = 0.9841). Thermodynamic parameters (ΔG < 0, ΔH < 0) indicated a spontaneous and exothermic process. Moreover, the adsorbent maintained good performance over three adsorption-desorption cycles, demonstrating reusability. These results demonstrate that combining PI, CS, UiO-66, and MoS_2_ offers a synergistic platform that addresses the shortcomings of the individual components and enables high-efficiency dye removal from wastewater.

**Supplementary Information:**

The online version contains supplementary material available at 10.1038/s41598-025-32760-x.

## Introduction

Synthetic organic dyes, widely applied for coloring textiles, paper, plastics, and food products, are recognized as toxic compounds. Industrial effluents containing these dyes therefore require effective treatment to ensure their safe removal^1^. MO, an anionic azo dye extensively used in the textile industry, is particularly concerning due to its toxicity and resistance to degradation, posing significant risks to human health, the environment, and aquatic life^2^. A variety of treatment strategies^3–9^ have been explored for dye removal (specially MO^10–18^) from contaminated water, among which adsorption, a widely used and highly efficient technique, has proven highly effective. This process involves the adherence of pollutants onto the surface of adsorbent materials and offers several advantages, including operational simplicity, cost-effectiveness, potential reusability of the adsorbent, and the avoidance of toxic byproduct formation^19–21^.

Nanofibers are increasingly used for dye adsorption from wastewater because they combine a high external surface-to-volume ratio, high porosity, fine pore structure, and possibility of surface functionalization, which together yield high adsorption capacity, rapid kinetics, good stability, and reusability^22–24^. Polyimide (PI) has been utilized as a polymer for dye removal applications due to its excellent properties such as high mechanical and thermal resistance, resistance to various solvents, and high formability^25–27^. If the base material lacks suitable functional groups, functionalization strategies, such as blending or chemical treatment, can be employed. Consequently, a wide variety of functional molecules have been incorporated into electrospun nanofibers to enhance their pollutant adsorption capabilities^28^. Chitosan (CS) has emerged as a promising candidate due to its amino and hydroxyl groups, which facilitate dye removal. It is cost-effective, hydrophilic, biocompatible and readily available. Although CS contains functional groups favorable for dye binding, its poor electrospinnability prevents the formation of high surface area structures, which limits its adsorption performance when used alone^29,30^.

Additionally, materials such as ZnO, TiO_2_, and molybdenum disulfide (MoS_2_) have demonstrated potential for removing pollutants from aqueous solutions. Among these, the two-dimensional (2D) MoS_2_ material is particularly promising due to its abundant active sites, chemical stability, and adjustable interlayer spacing^31–33^. However, a significant limitation of MoS_2_ lies in the limited availability of active sites, which results from Van der Waals interactions between layered crystal structures, causing the aggregation of MoS_2_ nanosheets. To address this challenge, researchers have explored the integration of metal-organic frameworks (MOFs) to enhance the properties of MoS_2_. MOFs are crystalline materials composed of metal ions and organic linkers, characterized by extensive porosity and well-defined pore structures^34–36^. These frameworks exhibit excellent environmental compatibility and low toxicity, making them ideal candidates for pollution removal applications. The coupling of MoS_2_ with MOF structures significantly improves pollutant adsorption efficiency in aqueous environments^37–39^. Among MOFs, UiO-66 is a highly stable material with a large specific surface area, excellent chemical and thermal stability, and a well-defined porous structure, making it widely applicable for dye removal from aqueous solutions through various methods, including adsorption^34^.

Recent studies have reported various adsorbents for MO removal; however, many of them operate under highly acidic/basic pH^40^, elevated temperatures^41^, or require difficult post-separation steps due to their powder form^42^. In addition, several reported materials involve complex or expensive synthesis procedures^43,44^. These limitations highlight the need for easily recoverable, water-stable hybrid adsorbents with practical operating conditions, which is the motivation of the present work. In this work, we designed and fabricated a novel nanofibrous adsorbent based on polyimide/chitosan@MoS_2_-UiO-66 (PI-CS@MoS_2_-UiO-66) fibers to overcome the inherent limitations of individual components. While CS provides abundant functional groups for dye adsorption, its poor electrospinnability restricts its direct use in nanofiber fabrication, and MoS_2_ suffers from nanosheet aggregation that reduces the available active surface area. To address these issues, electrospun PI fibers were employed as a robust, solvent-resistant support onto which CS@MoS_2_-UiO-66 particles were immobilized through a simple dip-coating and stabilization process. The UiO-66 framework effectively prevents MoS_2_ aggregation, while the surface deposition of CS on the PI nanofiber support improves the accessibility of functional groups and enhances the adsorption performance. Comprehensive characterization using XRD, BET, FT-IR, TGA, and SEM confirmed the successful integration of CS, MoS_2_, and UiO-66 onto the PI nanofiber matrix, significantly enhancing its adsorption capabilities. Systematic adsorption experiments revealed that the removal performance was strongly influenced by operational parameters such as adsorbent dosage, initial dye concentration, solution pH, and temperature. The adsorption efficiency increased at lower temperatures, indicating an exothermic nature of the process, and was pH-dependent, with maximum removal observed under neutral to slightly acidic conditions. The point of zero charge (pH_pzc_) of the adsorbent was determined using the pH drift method. Adsorption isotherm analysis showed that the Langmuir model best described the process, suggesting monolayer adsorption on a homogeneous surface. The maximum adsorption capacity ($\:{q}_{max}$) of 195.61 $\:\frac{\mathrm{m}\mathrm{g}}{\mathrm{g}}$ confirmed the high efficiency of the PI-CS@MoS_2_-UiO-66 nanofibers in removing MO. Thermodynamic studies revealed that the adsorption was spontaneous (negative ΔG), exothermic (negative ΔH), and accompanied by a decrease in system disorder (negative ΔS), supporting the feasibility of the process under standard environmental conditions. Furthermore, reusability tests demonstrated that the nanofibers retained high adsorption performance even after three consecutive cycles, highlighting their potential as efficient, stable, and reusable adsorbents for the removal of dyes from industrial wastewater.

## Materials and methods

### Materials

Polyimide (PI) was provided from Arkema Global, sodium chloride (NaCl), N,N-dimethylformamide (DMF, 99%), methyl orange (MO, C_14_H_14_N_3_NaO_3_S, 99%), acetic acid (CH_3_COOH, pure) and molybdenum disulfide (MoS_2_) were provided from Merck Chemical Co. and UiO-66, chitosan, glutaraldehyde, sodium hydroxide (NaOH, pure), hydrochloric acid (HCl) and ethanol (C_2_H_6_O, pure) were provided from local companies in Iran.

### Synthesis of PI nanofibers

The PI nanofibers were synthesized by electrospinning method. For this purpose, 0.1 g of PI was dissolved in 0.54 g DMF and stirred for 1.5 h at 800 rpm in the ambient temperature. Then the solution was transferred into a syringe and electrospinned. The optimal values of the electrospinning parameters were obtained by conducting several experiments. The rate of injection of 1.4 ml/h, supply voltage of 17 kV, distance between needle and collector of 15 cm and collector speed of 500 rpm were found as optimal conditions of electrospinning.

### Synthesis of PI/CS@MoS_2_-UiO-66

1.5 mg of UiO-66 and 1.5 mg of MoS_2_ were dispersed in 15 mL of acetic acid (2% v/v) and stirred for 30 min at 600 rpm in the ambient temperature. The solution was then ultrasonicated for 30 min. Next, 0.1 g CS was added to the solution and stirred for 30 min at 600 rpm in the ambient temperature. In the next stage, the electrospun PI fibers were dip coated in the solution for 20 min. At the end, the coated nanofibers took out from the solution and put into oven at 130 °C for 30 min to remove the extra solvent. The nanofibers were put into glutaraldehyde solution (1% v/v) for 20 min and finally washed several times with deionized water and let it to be dried to obtain PI-CS@MoS_2_-UiO-66 nanofibers.

###  Characterization

FT-IR spectra were recorded with a Tensor II (Bruker, Germany) in the range of wavenumber of 400–4000 $\:{\mathrm{cm}}^{-1}$. The level of crystallinity and structure of samples were determined by using a D8-Advance X-ray diffractometer (Bruker, Germany). For examining the morphology of the materials, a TESCAN-Vega3 scanning electron microscope (Czech) was employed. For the determination of MO removal, a Genesys 10vis UV-Vis spectrophotometer was employed. Thermogravimetric analysis was carried out using a TGA/DSC 1 thermogravimetric analyzer (Switzerland) to evaluate the thermal stability of the samples.

### MO adsorption test

The solution of MO was prepared in distilled water. The stock solution had a concentration of 400 ppm. Then dilution was performed with this solution. The absorbance spectra of MO was measured with a UV-vis spectrophotometer at wavelength 464 nm. The effects of various parameters such as pH, initial MO concentrations, adsorbent dosage, reusability, and temperature were studied. To investigate the effect of pH on the adsorption of MO, the initial pH of the MO solution was adjusted in the range of 5-9.5, by adding HCl (0.1 $\:\frac{\mathrm{mol}}{\mathrm{L}}$) and NaOH (0.1 $\:\frac{\mathrm{mol}}{\mathrm{L}}$) solutions, respectively. To test the influence of initial MO concentration, 15 mg of adsorbent was added into MO solution. The volume of solution was 30 mL and the MO concentration was between 20 and 400 ppm. In the investigation of adsorbent dosage, the amount of 5–35 mg of adsorbent was used. In the reusability section, 15 mg of the adsorbent was used to evaluate its performance over multiple cycles. For the regeneration process, the spent adsorbent was treated with a mixture of 20 mL of 0.1 N NaOH and 5 mL of ethanol, and the mixture was stirred for 60 min to desorb the adsorbed MO. The temperature effect on the adsorption of MO, was tested in the range of 20–40 °C. Finally, the point of zero charge (pH_pzc_) of the adsorbent was determined using the pH drift method.

## Results and discussion

### Characterization

**Fig. 1:**
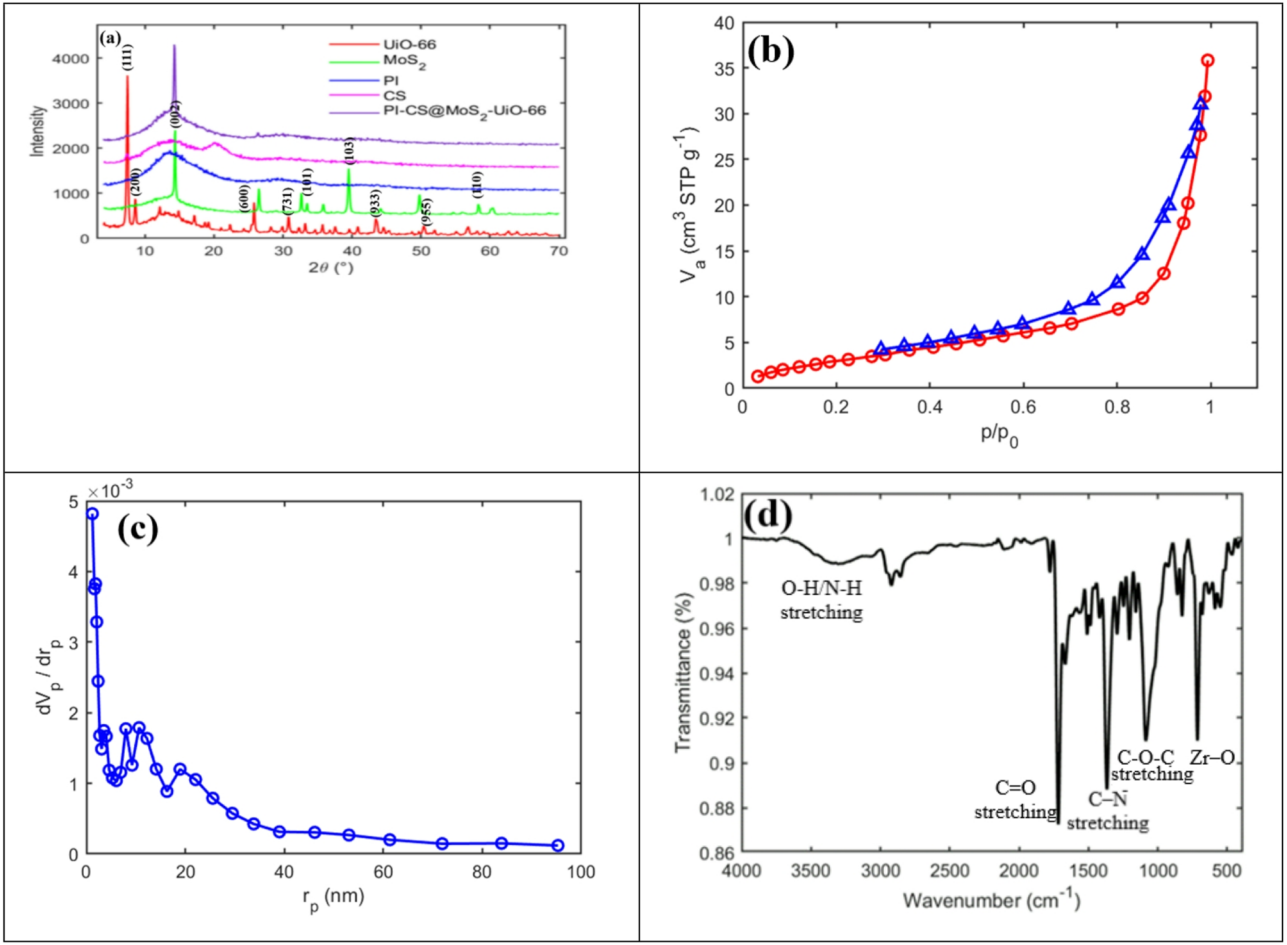
(**a**) XRD patterns of UiO-66, MoS_2_, PI, CS, and PI-CS@MoS_2_-UiO-66 nanofibers (**b**) N_2_ adsorption/desorption (**c**) pore ​​size distribution of PI-CS@MoS_2_-UiO-66 nanofiber and (**d**) FT-IR of PI-CS@MoS_2_-UiO-66 nanofiber before adsorption.

The XRD patterns of UiO-66, MoS_2_, PI, CS, and PI-CS@MoS_2_-UiO-66 nanofibers, as presented in Fig. 1a, offer valuable insights into the crystalline structures of the respective materials. The appearance of three distinct peaks at 2θ = 7.38°, 8.56°, and 25.67° confirms the presence of UiO-66 in its crystalline form, with no detectable impurities in its structure^45^. Similarly, three characteristic peaks for MoS_2_ are observed at 2θ values of approximately 14.5°, 33°, and 39.3°, indicating the presence of MoS_2_ in a crystalline structure^46^. As is well known, PI possesses an amorphous structure, which is also evident in the XRD pattern, showing a broad peak around 2θ = 15°^47^. In addition, CS also exhibits an amorphous structure, as observed in the XRD pattern, characterized by two broad peaks in the 2θ range of approximately 10° to 20°^48^. In the final PI-CS@MoS_2_-UiO-66 nanofibers, the overall pattern closely resembles the amorphous nature of PI due to the predominant presence of PI and CS. Moreover, the characteristic peak of MoS_2_ at 2θ = 14.5° is clearly identifiable, while the peak corresponding to UiO-66 at 2θ = 25.67° and at 2θ = 7.38° is also discernible, albeit to a lesser extent.

Figure 1b and c illustrates the N_2_ gas adsorption-desorption isotherm of the PI-CS@MoS_2_-UiO-66 nanofibers. According to the IUPAC classification, the adsorption isotherm for PI-CS@MoS_2_-UiO-66 nanofibers corresponds to type IV, while the hysteresis loop is categorized as type H3. The specific surface area and pore volume are 12.441 $\:\frac{{\mathrm{m}}^{2}}{\mathrm{g}}$ and 0.052099 $\:\frac{{\mathrm{cm}}^{3}}{\mathrm{g}}$, respectively. As shown in Table 1, the BET surface area of pure PI nanofibers is 20 $\:\frac{{\mathrm{m}}^{2}}{\mathrm{g}}$, which decreased to 12 $\:\frac{{\mathrm{m}}^{2}}{\mathrm{g}}$ after modification. This reduction is attributed to the coating of CS@MoS_2_-UiO-66 particles on the PI fibers, which partially blocked the pores of the PI nanofiber matrix. The results obtained from the BET test are presented in Table 1.

**Table 1:** BET results for MoS_2_, UiO-66, PI and PI-CS@MoS_2_-UiO-66 nanofiber.

Materials	BET surface area ($\:\frac{{\mathrm{m}}^{2}}{\mathrm{g}}$)	Average pore diameter (nm)	Total pore volume ($\:\frac{{\mathrm{cm}}^{3}}{\mathrm{g}}$)
MoS_2_	15	15.248	0.0582
UiO-66	1170	3.440	1.0062
PI Fiber	20	25.610	0.1306
PI-CS@MoS_2_-UiO-66 nanofiber	12	16.750	0.0521

The FT-IR test results for PI-CS@MoS_2_-UiO-66 nanofiber before MO adsorption is presented in Fig. 1d.

**Fig. 2:**
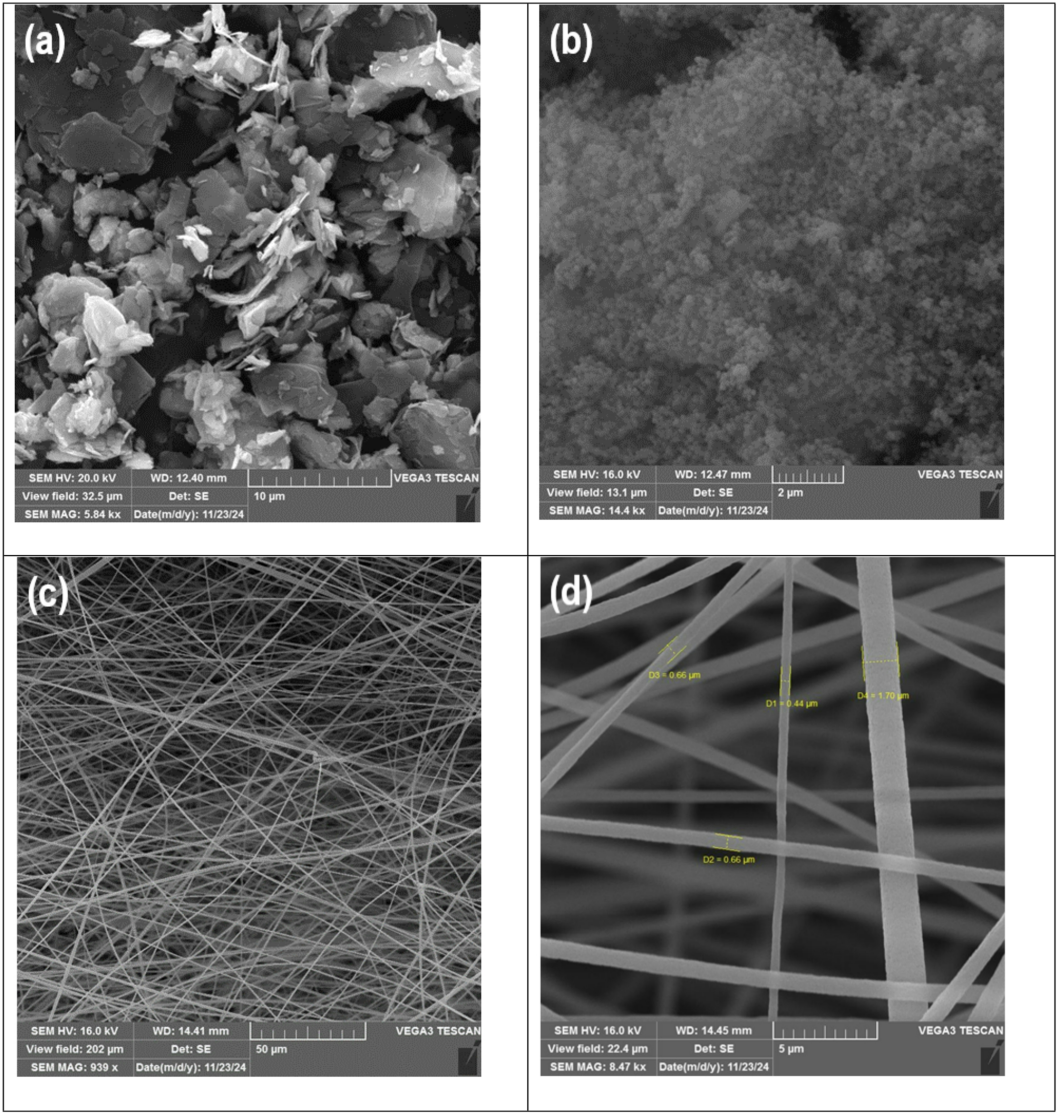

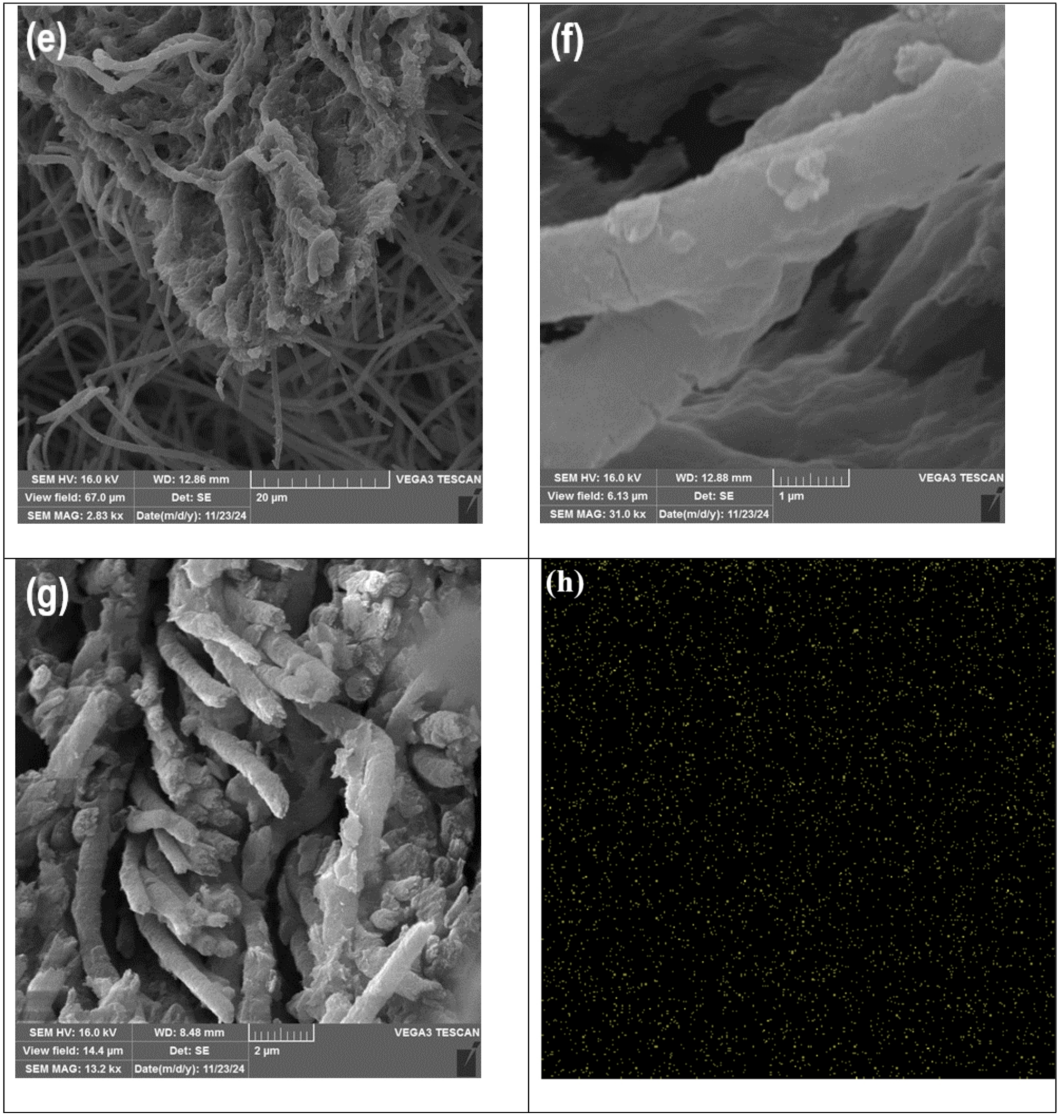

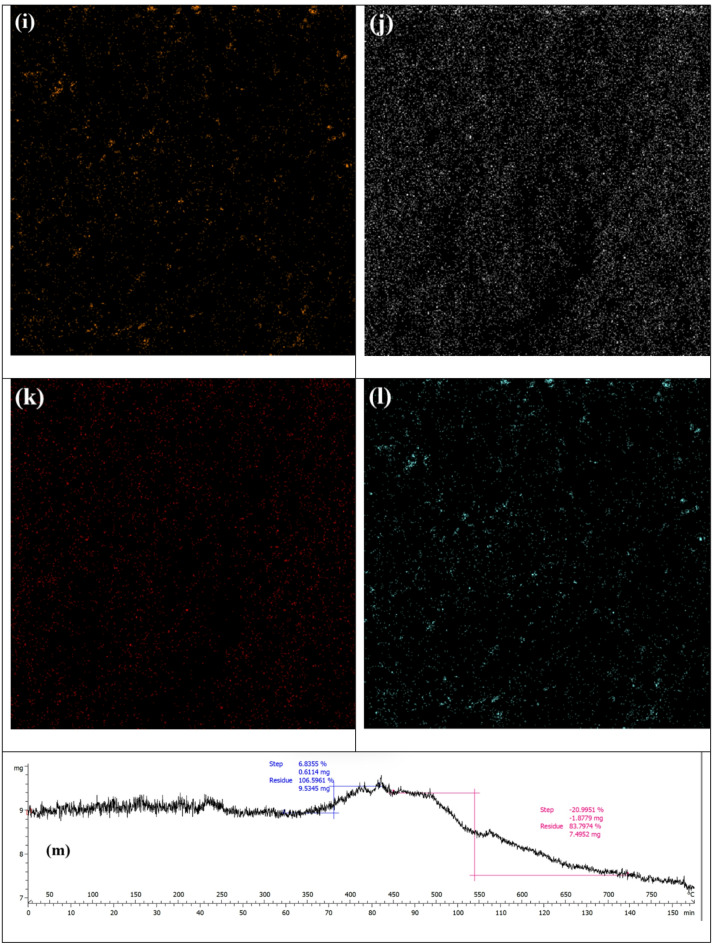

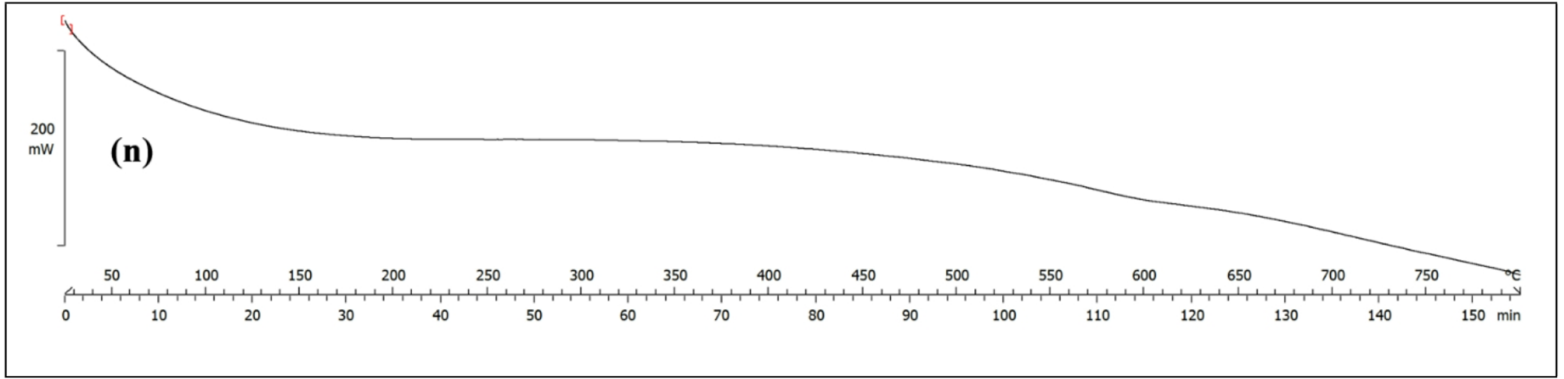
SEM of (**a**) MoS_2_ (**b**) UiO-66 (**c**) and (**d**) PI nanofiber (**e**) and (**f**) PI-CS@MoS_2_-UiO-66 nanofiber (**g**) cross-sectional view of the PI-CS@MoS_2_-UiO-66 nanofiber (**h**) to (**l**) mapping of Zr, Mo, C, O and S, respectively (**m**) and (**n**) TGA results of PI-CS@MoS_2_-UiO-66 nanofibers.

Figure 2 presents the SEM images of the components of the PI-CS@MoS_2_-UiO-66 nanofiber. The MoS_2_ nanosheets depicted in Fig. 2a, clearly show the layered structure. It is also evident that there is no MoS_2_ nanosheets aggregation, and a larger effective surface area is accessible. Some of these nanosheets exhibit thicknesses of less than a micrometer, reaching the nanoscale range. Figure 2b illustrates the UiO-66 particles, highlighting their morphological characteristics and uniform distribution. The observed particles appear to be in the micrometer range, with no large aggregates, which could indicate good dispersion and accessibility of the surface area for the adsorption. The particles are relatively uniform in size, which suggests a high degree of homogeneity. Figure 2c,d display the electrospun PI nanofibers, which exhibit a well-formed and uniform structure without any visible bead formation. The good electrospinning process yields fibers with a high surface area for adsorption, and the image supports this high surface area. Additionally, the fiber diameter ranges approximately between 450 nm and 1.7 μm. Figure 2e and f illustrate the integration of MoS_2_ and UiO-66 nanomaterials onto the CS-coated PI nanofibers, demonstrating a well-attached and uniformly distributed structure. The strong adhesion of the nanomaterials to the fibers is clearly evident in the images. There is no particle aggregation, and a good distribution of particles is observed on the fiber surface, which allows for maximum utilization of the material’s capacity for adsorption. Additionally, Fig. 2g presents the cross-sectional view of the PI-CS@MoS_2_-UiO-66 nanofibers, providing further insights into their internal morphology. In Fig. 2h-l, the mappings of zirconium, molybdenum, carbon, oxygen, and sulfur are presented, indicating the presence of these elements and their uniform distribution within the final nanofibers.

Thermogravimetric and calorimetric analyses of the PI-CS@MoS_2_-UiO-66 nanofibers were carried out under nitrogen, and the results are presented in Fig. 2m and n. The fibers remained highly stable up to 250 °C, consistent with the inherent thermal resistance of PI. In the range of 250–300 °C, CS is expected to begin thermal degradation, which would typically result in mass loss^49^. Interestingly, however, the sample exhibited a slight anomalous weight gain (6.8%) around 350–400 °C. This unexpected increase may arise from several factors, such as trace oxygen contamination, buoyancy effects of the instrument, or, as an additional possibility, local oxidation of MoS_2_ or UiO-66 induced by oxygenated fragments released during CS decomposition.

The main weight loss (21%) occurred between 400 and 650 °C, which corresponds to the progressive decomposition of CS and the onset of PI and UiO-66 degradation^50,51^. Around 500 °C, the breakdown of the PI backbone and collapse of the UiO-66 framework become evident. At higher temperatures (700 °C), MoS_2_ begins its gradual decomposition, which continues more slowly up to 800°C^52^. Beyond this point, the weight curve flattens, indicating the formation of a thermally stable residue.

Overall, the composite demonstrates remarkable thermal stability up to 350 °C and maintains a high char yield (84%) at 800 °C, reflecting the stabilizing role of the PI matrix together with the inorganic phases that preserve structural integrity under elevated temperatures.

### MO adsorption

In this experimental investigation, we employed a new PI-CS@MoS_2_-UiO-66 nanofiber that adsorbs MO dye from wastewater. The CS and UiO-66 functional groups, along with the high surface area and porosity provided by MoS_2_ and UiO-66, enable efficient adsorption of the MO dye molecules onto the nanofibers. Among the tested loadings of MoS_2_ and UiO-66 relative to CS (1, 3, and 5 wt%), the 3 wt% formulation exhibited the best adsorption performance; therefore, it was selected for subsequent experiments. In this section the effect of various parameters on adsorption efficiency is examined. When a fixed initial concentration was required, it was selected at a level (e.g., 100 or 200 ppm) that ensured the adsorbent would not completely remove MO within a short period, allowing the adsorption process to proceed over the full 3 h duration and enabling reliable kinetic and equilibrium analysis. The mean equilibrium adsorption capacity (q_e_, $\:\frac{\mathrm{m}\mathrm{g}}{\mathrm{g}}$) for PI, MoS_2_, CS, and UiO-66 was determined to be 6, 21.4, 33.24 and 73.15 $\:\frac{\mathrm{m}\mathrm{g}}{\mathrm{g}}$, respectively.

#### Effect of dosage of adsorbent on the MO adsorption

**Fig. 4:**
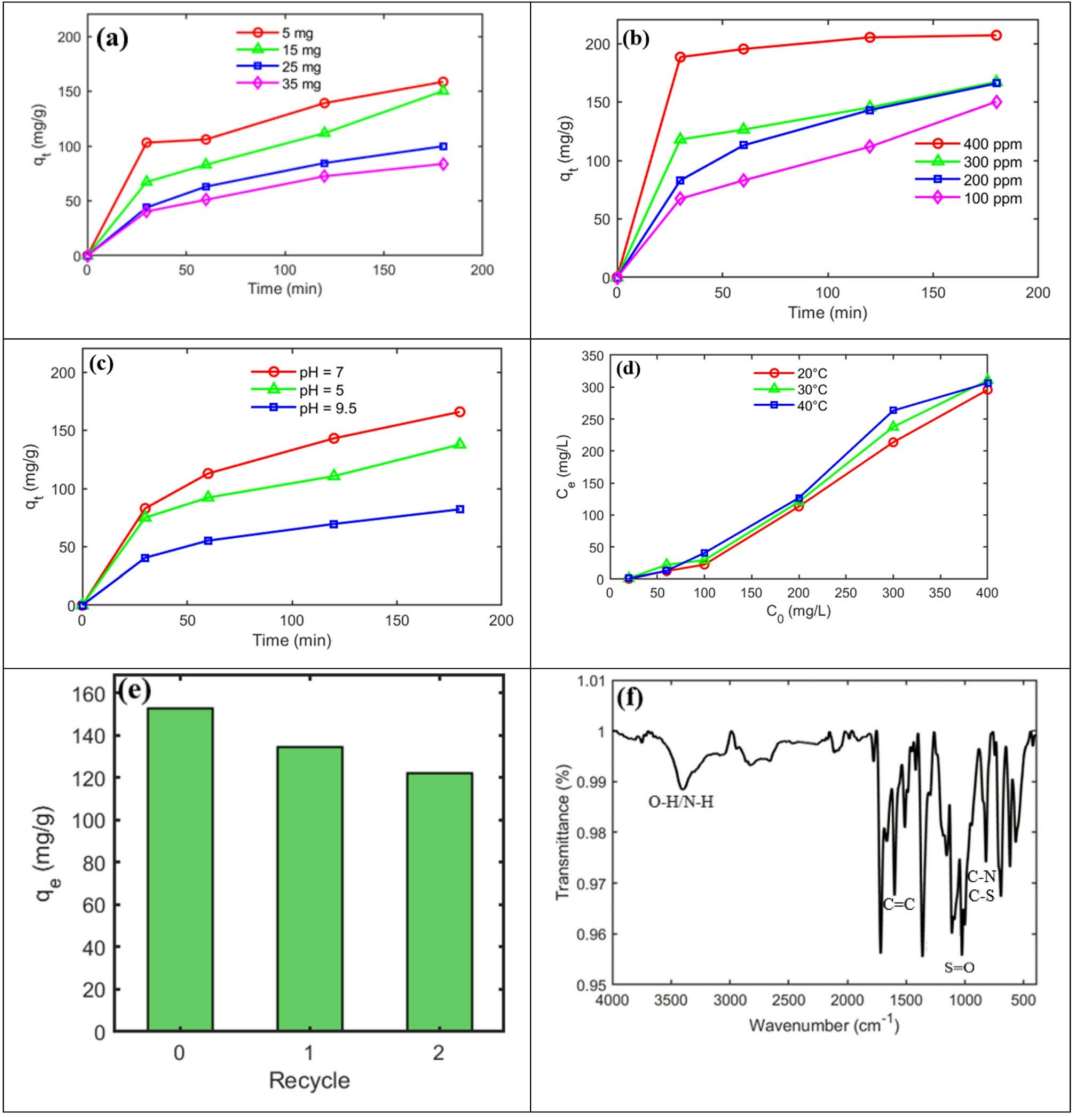
(**a**) The effect of dosage of adsorbent on the removal of MO dye by PI-CS@MoS_2_-UiO-66 nanofiber (**b**) The effect of initial concentration of MO on the removal of MO by PI-CS@MoS_2_-UiO-66 nanofiber (**c**) The effect of pH of solution on the removal of MO by PI-CS@MoS_2_-UiO-66 nanofiber (**d**) The effect of temperature on the removal of MO by PI-CS@MoS_2_-UiO-66 nanofiber (**e**) Reusability of PI-CS@MoS_2_-UiO-66 nanofiber (**f**) FT-IR of PI-CS@MoS_2_-UiO-66 nanofiber after adsorption.

The adsorbent amount plays an important role in dye adsorption. Solutions with an MO concentration of 100 $\:\frac{\mathrm{mg}}{\mathrm{L}}$ and a volume of 30 mL at pH = 7 were used in this study. The adsorbent doses were 5, 15, 25 and 35 mg. In Fig. 4a the effect of different doses of adsorbent on the MO removal was investigated as a function of time. The overall trend indicates that cumulative adsorption increased with time for all doses. The adsorption rate was higher during the initial phase (first 30 min), which can be attributed to the abundance of available active sites at the beginning of the process. At lower adsorbent dosages, the mean capacity of adsorption (q, $\:\frac{\mathrm{m}\mathrm{g}}{\mathrm{g}}$) was higher because the adsorption sites were exposed to a greater number of MO molecules and were occupied more rapidly. Over time, the rate decreased due to repulsive interactions among the adsorbed MO molecules and the gradual saturation of adsorption sites, leaving fewer active sites available for further uptake.

#### Effect of initial MO concentration on the adsorption

In this work, different initial concentrations 100, 200, 300 and 400 $\frac{\mathrm{mg}}{\mathrm{L}}$ of MO were examined in a solution with volume of 30 mL at pH = 7 and an adsorbent amount of 15 mg. As shown in Fig. 4b, cumulative adsorption increased with time at all initial concentrations. The adsorption rate was faster during the first 30 min due to the abundance of available active sites, after which it gradually decreased as the sites became occupied. At higher initial concentrations, the adsorption capacity was greater because more MO molecules were available to interact with the adsorbent. At 400 ppm, nearly all adsorption sites were rapidly saturated, and the MO concentration remained almost unchanged thereafter.

#### Effect of pH on the MO adsorption

To investigate the effect of pH on the adsorption of MO, the pH was varied in the range of 5–9.5, which includes acidic, neutral and basic regions. The initial concentration of MO was 200 $\:\frac{\mathrm{mg}}{\mathrm{L}}$, the solution volume was 30 mL and the amount of adsorbent was 15 mg. As shown in Fig. 4c, the adsorption of MO was higher in acidic regions than in basic regions. MO is an anionic dye containing sulfonate (–SO_3_^−^) and nitrogen functional groups, which explains its higher adsorption in neutral and slightly acidic environments compared to basic conditions. As shown in Fig. 4c, adsorption was greatest at neutral pH, where the nanofiber surface exhibited optimal charge distribution and active adsorption sites. In slightly acidic media, the adsorbent surface is negatively charged. However, the excess H^+^ ions present in acidic solution tend to attach to the negatively charged sites of the nanofiber, thereby competing with MO anions and reducing the adsorption capacity compared to neutral pH. Under basic conditions, the stronger negative charge of both the adsorbent surface and MO, together with OH^−^ ions in solution, generates strong electrostatic repulsion, resulting in significantly reduced adsorption.

#### Effect of temperature on the MO adsorption

The effect of temperature on MO adsorption was also investigated using a 30 mL of solution with 15 mg of adsorbent and initial different concentrations of MO. Three different temperatures of 20, 30, and 40 °C were examined for a period of 3 h. As it is shown in Fig. 4d, adsorption occurred better at lower temperatures. These results indicate that the adsorption of MO on the adsorbent is exothermic. The observed higher adsorption efficiency of MO at lower temperatures can be attributed to the reduced kinetic energy of the dye molecules, favorable thermodynamic conditions, and stable interactions between the MO molecules and the functionalized nanofiber surface.

#### Reusability

The reusability of an adsorbent is of significant importance in both environmental and economic contexts. Repeated use of these adsorbents minimizes the need for the continuous production of new materials, thereby reducing waste generation and resource consumption. Furthermore, the ability to regenerate and reuse fiber-based adsorbents enhances the efficiency of dye removal from wastewaters. This sustainable approach offers a promising solution for improving the overall sustainability of the textile industry wastewater treatment. In this section, 15 mg of MO saturated nanofibers was immersed in a solution containing 20 mL NaOH 0.1 N and 5 mL of pure ethanol for 60 min. Then, the adsorption process of MO was reperformed. This procedure was repeated twice, and the results are presented in the Fig. 4e. The results indicate that after three cycles of MO adsorption and desorption using the prepared solution, the fibers still maintain good performance.

#### Plausible adsorption mechanism

The FT-IR test results for PI-CS@MoS_2_-UiO-66 nanofiber after MO adsorption is presented in Fig. 4f. A comparison of the spectra before and after adsorption indicates the emergence of a new peak at approximately 1600 $\:{\mathrm{cm}}^{-1}.$ A strong peak in this region suggests the presence of aromatic rings from MO interacting with the PI-CS@MoS_2_-UiO-66 nanofiber surface^53^. Although FT-IR does not directly confirm the presence of π–π stacking, considering the aromatic structures of both UiO-66 and MO, such interactions are highly plausible and are likely to play an important role in the overall adsorption mechanism. The N-H and O-H interactions (3000–3800 $\:{\mathrm{cm}}^{-1}$) suggest hydrogen bonding between chitosan and MO during adsorption. The change in the intensity of the 618 $\:{\mathrm{cm}}^{-1}$ peak may be attributed to UiO-66, where a bond forms between the carboxyl chains or Zr-O…$\:{SO}_{3}^{-},$ linking MO to the UiO-66 surface. The FT-IR spectra before and after adsorption confirm this interaction, with key peaks appearing in the 1100–1300 $\:{\mathrm{cm}}^{-1}$ range, corresponding to S=O bonds, as well as possible Mo-S…$\:{SO}_{3}^{-}$ interactions^54,55^. The increased intensity of the 822 $\:{\mathrm{cm}}^{-1}$ peak after MO adsorption is most likely attributed to π-π interactions between the aromatic systems of MO and PI. These interactions can induce changes in out-of-plane C-H bending vibrations, which are observed in the 800–850 $\:{\mathrm{cm}}^{-1}$ range^56^.

#### Point of zero charge

**Fig. 5:**
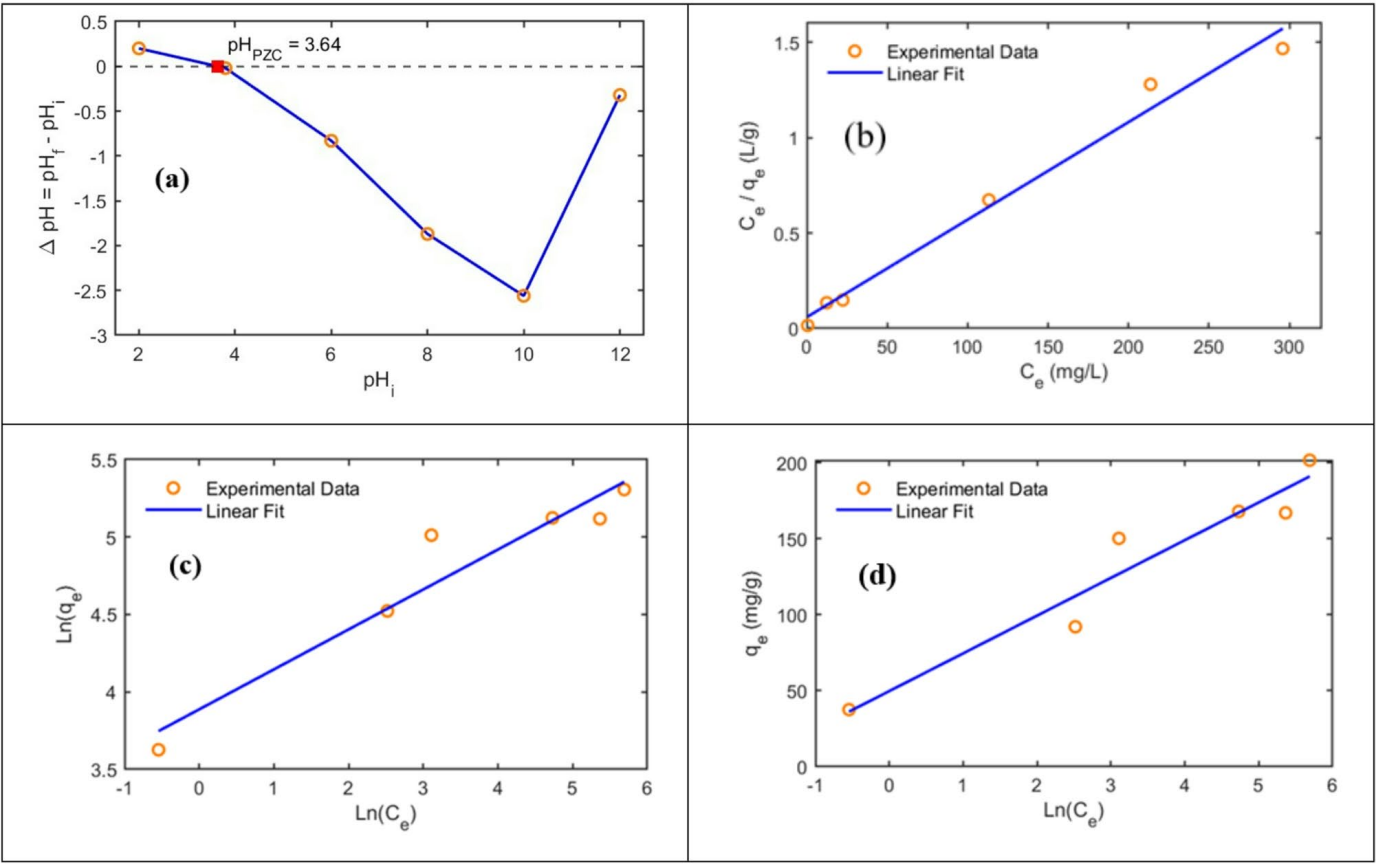
(**a**) pH_pzc_ of the PI-CS@MoS_2_-UiO-66 nanofiber (**b**) Langmuir (**c**) Freundlich and (**d**) Temkin model fits for the adsorption of MO onto the PI-CS@MoS_2_-UiO-66 nanofiber in 20 °C.

The pH_pzc_ of the PI-CS@MoS_2_-UiO-66 nanofibers was determined using the pH drift method. A 0.01 M NaCl solution was prepared and divided into six 50 mL portions, and the initial pH values were adjusted to 2, 3.8, 6, 8, 10, and 12 using 1 M HCl and 1 M NaOH solutions. Subsequently, 5 mg of the adsorbent was added to each solution, and the mixtures were shaken for 24 h to reach equilibrium. After equilibration, the final pH of each solution was recorded. The pH_pzc_ was identified as the point where the initial and final pH values were equal (ΔpH = pH_f_ – pH_i_ = 0). The surface of the adsorbent is positively charged at pH values below the pH_pzc_, favoring the adsorption of anionic dye molecules, while it becomes negatively charged above this point, which can influence the adsorption mechanism. The pH_pzc_ of the PI-CS@MoS_2_-UiO-66 nanofibers was found to be 3.64, indicating the pH at which the net surface charge is zero. In this study, the pH range investigated for adsorption (5.0–9.5) lies entirely above the pH_pzc_, meaning the nanofiber surface remains negatively charged throughout the tested conditions. Although pH values below 3.64 would generate a positively charged surface that is favorable for anionic dyes such as MO, such highly acidic conditions fall outside realistic operational ranges for practical wastewater treatment. Therefore, the adsorption behavior was evaluated only under near neutral conditions, which better represent real world application scenarios. The pH drift curve used to determine this parameter is shown in Fig. 5a.

### Adsorption isotherms

The data obtained in this work, have been plotted based on the four models, and the various parameters of the formulas for these models have been determined. The isotherm models (Langmuir, Freundlich, and Temkin) are presented in the Supplementary Information (S.1). The results are presented in Fig. 5b-d; Table 2.

As shown in Table 2, the data from this experiment exhibit a better fit to the Langmuir model. According to the results, the adsorption equilibrium data showed the best fit with the Langmuir isotherm model. The excellent linear correlation indicates that the adsorption process follows a monolayer coverage on a homogeneous surface with identical and energetically equivalent active sites. This finding suggests that once a dye molecule occupies a binding site, no further adsorption can occur at that location, supporting the assumption of uniform adsorption energy across the surface. The Langmuir model also allowed the theoretical determination of the maximum adsorption capacity ($\:{q}_{max}$), which provides a key parameter for evaluating the adsorption potential of adsorbent in wastewater treatment applications.

**Table 2:** The isotherm constants of adsorption models for MO adsorption

Langmuir			Freundlich			Temkin		
${q}_{max}$ ($\frac{\mathrm{mg}}{\mathrm{g}}$)	${K}_{L}$ ($\frac{\mathrm{L}}{\mathrm{mg}}$)	*R* ^2^	${K}_{F}$ ($\frac{\mathrm{mg}}{\mathrm{g}}$)	N	*R* ^2^	B ($\frac{\mathrm{J}}{\mathrm{mol}}$)	${K}_{T}$ ($\frac{\mathrm{L}}{\mathrm{g}}$)	*R* ^2^
195.6102	0.0864	0.9841	48.7987	3.8751	0.9260	24.8232	7.4296	0.9271

### Adsorption thermodynamics

Thermodynamic studies are crucial for understanding the adsorption process. In this section, the thermodynamic parameters of adsorption, including Gibbs free energy change (ΔG), enthalpy change (ΔH), and entropy change (ΔS), were determined based on equilibrium data at three different temperatures: 20, 30, and 40 °C. The thermodynamic models and derivations are presented in the Supplementary Information S.2. The corresponding thermodynamic parameters are presented in Table 3.

**Table 3:** Thermodynamic parameters for the adsorption of MO dye onto PI-CS@MoS_2_-UiO-66 nanofiber.

T (°C)	ΔG ($\:\frac{\mathrm{k}\mathrm{J}}{\mathrm{m}\mathrm{o}\mathrm{l}}$)	ΔH ($\:\frac{\mathrm{k}\mathrm{J}}{\mathrm{m}\mathrm{o}\mathrm{l}}$)	ΔS ($\:\frac{\mathrm{k}\mathrm{J}}{\mathrm{m}\mathrm{o}\mathrm{l}\:\mathrm{K}}$)
20	− 3.18682	− 19.64	− 0.05660
30	− 2.17543		
40	− 2.07480		

Since all values of ΔG are negative, the adsorption process is spontaneous at all studied temperatures. However, the increasing magnitude of ΔG (less negative at higher temperatures) suggests that the spontaneity of adsorption decreases with temperature, indicating a preference for adsorption at lower temperatures. The negative enthalpy change (ΔH) confirms that the adsorption process is exothermic, meaning heat is released as adsorption occurs; this indicates that adsorption occurs more effectively at lower temperatures. The negative entropy change (ΔS) suggests that the adsorption process leads to a decrease in system disorder, which is expected as adsorbate molecules become more structured when bound to the adsorbent surface.

To provide a broader context for the present study, a comparison with previously reported adsorbents based on individual components of this work (PI, CS, MoS_2_, and UiO-66) has been included. As summarized in Table 4, the adsorption capacities in the literature for these materials are listed alongside the results of this study. This comparative overview helps to place the current work within the broader research landscape. It should be noted that this table does not encompass all studies on MO adsorption, as a wide variety of other adsorbents with different compositions and structures have also been reported.

**Table 4:** Comparison of previously reported adsorbents based on PI, CS, MoS_2_, and UiO-66 for MO removal with the present study.

Adsorbent name	$\:{q}_{max}$ ($\:\frac{\mathrm{m}\mathrm{g}}{\mathrm{g}}$)	References
Amino groups functionalized UiO-66 MOF	148.40	^42^
CS/graphene oxide/gelatin hydrogel beads	257.20	^57^
Quaternized magnetic CS	486.13	^58^
Thiolated CS	434.89	^59^
Magnetic recyclable Fe_3_O_4_ /CS-DTPA	1541.50	^60^
CCM/TiO_2_@NC membranes	661.40	^61^
Hollow MoS_2_ (h-MoS_2_) microspheres	41.52	^55^
CS/UiO-66	900.00	^62^
Goethite impregnated with CS beads	84.00	^63^
CS schiff bases	20	^64^
PI-CS@MoS_2_-UiO-66 nanofiber	195.61	This work

## Conclusion

In this study some PI-CS@MoS_2_-UiO-66 nanofiber as an effective adsorbent for the removal of MO dye from aqueous solutions were successfully synthesized and characterized. The characterization results, including XRD, BET, FT-IR, TGA and SEM, confirmed the successful integration of CS, MoS_2_, and UiO-66 onto the PI nanofiber matrix, enhancing its adsorption capabilities. Experimental investigations revealed that the adsorption process was influenced by various factors, including adsorbent dosage, initial dye concentration, solution pH, and temperature. The results indicated that adsorption efficiency was higher at lower temperatures, confirming an exothermic adsorption process. Additionally, the adsorption performance was pH-dependent, with optimal adsorption occurring in neutral and slightly acidic conditions. Adsorption isotherm studies demonstrated that the experimental data were best fitted to the Langmuir isotherm model, suggesting monolayer adsorption on a homogeneous surface. The maximum adsorption capacity (q_max_) of 195.61 mg/g further confirms the high efficiency of PI-CS@MoS_2_-UiO-66 in dye removal. Thermodynamic parameters revealed that the adsorption was spontaneous (negative ΔG), exothermic (negative ΔH), and led to a decrease in system disorder (negative ΔS), supporting the feasibility of the process under standard environmental conditions. Finally, reusability experiments demonstrated that the nanofiber retained good adsorption capacity even after three cycles, indicating its potential for reuse with acceptable performance. Overall, the PI-CS@MoS_2_-UiO-66 nanofiber demonstrates promising potential as a high-performance and efficient adsorbent for the removal of MO.

## Supplementary Information

Below is the link to the electronic supplementary material.

Supplementary Material 1

## Data Availability

The data that support the findings of this study are available from the corresponding author upon reasonable request. Requests for materials, data, or additional information should be directed to Vahid Hemmati (vah.hemmati@gmail.com).
