# Supplementary material for: High-efficiency removal of methyl orange from wastewaters using polyimide/chitosan-MoS2-UiO-66 nanofiber adsorbents
Source: Sci Rep. 2025 Dec 17;16:2929. doi: 10.1038/s41598-025-32760-x (PMC12827241; doi:10.1038/s41598-025-32760-x)
Supplement: Supplementary file 1 — Supplementary Material 1 [file 41598_2025_32760_MOESM1_ESM.docx]

Supplementary data for:

High-Efficiency Removal of Methyl Orange from Wastewaters Using Polyimide/Chitosan-MoS_2_-UiO-66 Nanofiber Adsorbents

Vahid Hemmati^a *^, Gholamreza Karimi^a^, Dariush Mowla^a b^

^a^ Chemical Engineering Department, School of Chemical and Petroleum Engineering, Shiraz University, Shiraz 7134851154, Iran

^b^ Environmental Research Center in Petroleum and Petrochemical Industries, School of Chemical and Petroleum Engineering, Shiraz University, Shiraz 7134851154, Iran

^*^ Corresponding author. Tel.: +98 71 36473170. E-mail addresses: Vah.hemmati@gmail.com

**S. 1. Isotherm study**

The equilibrium capacity of adsorption (*q_e_*, $\frac{\mathrm{mg}}{g}$) was obtained using the following equation:

$q_{e}$= $\frac{C_{0}-C_{e}}{m}$ × *V* (1)

In this equation, $C_{0}$ is the initial concentration and $C_{e}$ is the equilibrium concentration of MO ($\frac{\mathrm{mg}}{L}$), *m* (g) is the adsorbent mass and *V* (L) is the MO solution volume.

Adsorption isotherm is a crucial aspect of understanding the adsorption processes in various scientific and industrial applications. Isotherms provide valuable insights into the interaction between the adsorbate and the adsorbent. By analyzing isotherm models, researchers can optimize conditions for maximum adsorption efficiency and better understanding the adsorption mechanism. The four common methods for studying adsorption isotherms are the Langmuir, Freundlich, Dubinin-Radushkevich and Temkin models. These four methods are explained here and then the data obtained for this research work were examined to fit which method.

The Langmuir isotherm model assumes monolayer adsorption onto a surface with a finite number of identical sites. The linearized Langmuir isotherm is represented by the equation:

$\frac{C_{e}}{q_{e}}$ = $\frac{1}{K_{L}q_{max}}$ + $\frac{C_{e}}{q_{max}}$ (2)

where $q_{e}$ is the amount of adsorbate adsorbed per unit mass of adsorbent, $q_{max}$ is the maximum adsorption capacity, $K_{L}$ is the Langmuir constant, and $C_{e}$ is the equilibrium concentration of adsorbate in solution.

The Freundlich isotherm model is an empirical equation that describes adsorption on heterogeneous surfaces with different types of adsorption sites. It is expressed as:

ln $q_{e}$= ln $K_{F}$ + $\frac{1}{n_{F}}$ ln $C_{e}$ (3)

where $q_{e}$ is the amount of adsorbate adsorbed per unit mass of adsorbent, $K_{F}$is the Freundlich constant indicative of the adsorption capacity, $C_{e}$ is the equilibrium concentration of adsorbate in solution, and $\frac{1}{n_{F}}$ is a dimensionless parameter indicating the adsorption intensity or surface heterogeneity.

The Dubinin-Radushkevich (D-R) isotherm model is used to describe adsorption processes with a Gaussian energy distribution on heterogeneous surfaces. It is particularly useful for distinguishing between physical and chemical adsorption. The linearized D-R isotherm is given by:

ln $q_{e}$ = ln $q_{max}$ – B$ℇ^{2}$ (4)

where $q_{e}$ is the amount of adsorbate adsorbed per unit mass of adsorbent, $q_{max}$ is the maximum adsorption capacity, B is a constant related to the adsorption energy, and ℇ is the Polanyi potential, defined as:

ℇ = RT ln (1+$\frac{1}{C}$) (5)

where *R* is the gas constant and *T* is the absolute temperature.

The Temkin isotherm model assumes that the heat of adsorption decreases linearly with coverage due to adsorbate-adsorbent interactions. It is expressed as:

$q_{e}$ = $\frac{RT}{B_{T}}$ ln ($A_{T}C_{e}$) (6)

where $q_{e}$ is the amount of adsorbate adsorbed per unit mass of adsorbent, $A_{T}$ is the Temkin isotherm constant, $B_{T}$ is a constant related to the heat of adsorption, and $C_{e}$ is the equilibrium concentration of adsorbate in solution.

The capacity of adsorption (*q*, $\frac{\mathrm{mg}}{g}$) at any time, was obtained using the following equation:

*q* = $\frac{C_{0}-C}{m}$ × *V* (7)

Where $C_{0}$is initial concentration and $C$ is the concentration of MO ($\frac{\mathrm{mg}}{L}$) at any specific time, *m* (g) is the adsorbent mass and *V* (L) is the MO solution volume^1^.

**S. 2. Thermodynamic study**

The equations of thermodynamic adsorption are:

$K_{c}$ = $\frac{q_{e}}{C_{e}}$ (8)

ln $K_{c}$ = $\frac{\Delta S}{R}$ - $\frac{\Delta H}{RT}$ (9)

ΔG = $\Delta H$ - T $\Delta S$ (10)

Where $K_{c}$ is the distribution coeﬃcient, $q_{e}$ is the adsorption capacity, $C_{e}$ is the solution concentration at the equilibrium state, ΔG ($\frac{\mathrm{kJ}}{\mathrm{mol}}$) is the Gibbs free energy, R (0.008314 $\frac{\mathrm{kJ}}{mol K}$) is the gas constant, T (K) is the absolute temperature, ΔS ($\frac{\mathrm{kJ}}{mol K}$) is the entropy change and ΔH ($\frac{\mathrm{kJ}}{\mathrm{mol}}$) is the enthalpy change^2^.

**References:**

1. Karim, K. Copper adsorption Behavior in some Calcareous Soils using Langmuir, Freundlich, Temkin, and Dubinin-Radushkevich Models. *J. Soil Sci. Agric. Eng.* **11**, 27–34 (2020).

2. El Jery, A. *et al.* Isotherms, kinetics and thermodynamic mechanism of methylene blue dye adsorption on synthesized activated carbon. *Sci. Rep.* **14**, 970 (2024).
